# Household air pollution: another threat to Indigenous people in Brazil

**DOI:** 10.36416/1806-3756/e20250318

**Published:** 2025-09-22

**Authors:** Rafael Futoshi Mizutani, Ubiratan de Paula Santos

**Affiliations:** 1. Divisão de Pneumologia, Instituto do Coracao - InCor - Hospital das Clinicas, Faculdade de Medicina, Universidade de Sao Paulo - HCFMUSP - Sao Paulo (SP) Brasil.

Household air pollution (HAP) is a significant health risk worldwide. According to the 2021 Global Burden of Disease study, 33.8% of the global population is exposed to high levels of HAP, contributing to 111 million disability-adjusted life years-or 3.9% of all disability-adjusted life years-and resulting in 3.11 million deaths.^1^ The impact of HAP is disproportionate, with the greatest burden falling on the lowest socioeconomic regions of Africa, Asia, and Latin America, especially in remote communities.^1^ ^,^ ^2^

HAP is primarily caused by the burning of materials such as wood, coal, charcoal, crop residues, and animal feces for cooking and heating.^3^ Vulnerable groups such as women, children, and the elderly are the most affected because they spend more time indoors than do men.^4^ These groups are also more susceptible to the adverse health effects of air pollution. In pregnant women, HAP increases the risk of preterm births and low birth weight.^1^ Children exposed to HAP are at a heightened risk of respiratory infections, asthma, impaired lung development, and even death. In 2021, HAP was responsible for 500,000 deaths among children under five years of age, accounting for 11% of global under-five mortality.^1^ Among the elderly, HAP is a major risk factor for COPD, cardiovascular disease, lung cancer, diabetes, and premature death.^1^

Despite extensive research on the morbidity and mortality of HAP, directly measuring exposure remains a challenge, as highlighted by Gioda^5^ in a recent study published in the *Jornal Brasileiro de Pneumologia*. In the study, Gioda measured fine particulate matter levels inside traditional Indigenous dwellings (*malocas*) in various Amazonian communities over short periods (of 20-60 min), comparing the indoor measurements with those taken outdoors. Limited access to power sources restricted the duration of the measurements. Although the mean fine particulate matter levels during firewood burning were recorded at 203 ± 261 µg/m^3^, it is likely that mean daily exposure is lower, given the intermittent nature of fire use. Nevertheless, the study demonstrates that Indigenous people are regularly exposed to high levels of HAP. 

Indigenous populations in Brazil face numerous socioeconomic challenges in comparison with non-Indigenous groups. They experience limited access to education, health care, electricity, and nutritious food, leading to higher childhood mortality rates and lower life expectancy.^6^ In addition, record-breaking wildfires in the Amazon have compounded their exposure to outdoor air pollution, further threatening their health.^7^ ^,^ ^8^ Deforestation, illegal mining, and the shifting weather patterns caused by climate change have led to more frequent famines, further exacerbating their vulnerability.^9^

Addressing these health disparities requires concerted efforts from governmental bodies. It is essential that the government provide full support to improve the living conditions of Indigenous communities, improving housing ventilation and stove exhaust; providing cleaner energy sources (such as gas); and expanding access to health services. Protecting Indigenous territories from deforestation, forest burning, illegal occupation, and mining is also critical, as is improving food security for these communities. The Brazilian society owes a significant debt to its Indigenous peoples. Closing the gap in these inequalities is vital to building a more just and equitable society for all. 
